# Association between NF-*κ*B Signal Pathway-Related Gene Polymorphisms and Response to Alendronate Treatment in Postmenopausal Chinese Women with Low Bone Mineral Density

**DOI:** 10.1155/2022/2461716

**Published:** 2022-03-24

**Authors:** Xiaoyi Shen, Sasa Tan, Xianzhen Feng, Wenzhen Fu, Yunqiu Hu, Miao Li, Wenjie Wang, Hu Yuan, Li Liu, Chun Wang, Fei Hua

**Affiliations:** ^1^Department of Endocrinology, The Third Affiliated Hospital of Soochow University, 185, Juqian Street, Changzhou 213003, China; ^2^Shanghai Clinical Research Center of Bone Disease, Department of Osteoporosis and Bone Disease, Affiliated Sixth People's Hospital, Shanghai Jiao Tong University, 600 Yishan Road, Shanghai 200233, China; ^3^Department of General Practice, Tongren Hospital, Shanghai Jiao Tong University School of Medicine, 111 Xianxia Road, Shanghai 200336, China

## Abstract

**Background:**

Osteoporosis is a systemic bone disease characterized by reduction of bone content. Bisphosphonates are first-line treatments for osteoporosis, but they have variable effectiveness. Genetic factors may explain these differences. The NF-*κ*B signaling pathway plays a key role in the regulation of bone metabolism. We aimed to determine whether genetic variations in the NF-*κ*B signaling pathway affect the effectiveness of alendronate in postmenopausal Chinese women with low bone mass.

**Methods:**

We recruited 455 postmenopausal Han Chinese women with primary osteoporosis or osteopenia aged 48–90 yrs who had experienced no spontaneous menses for at least 1 yr. All participants had dual X-ray absorptiometry (DEXA) bone mineral density (BMD) measurement at baseline and 1 yr after treatment. Treatment involved 1 yr administration of 70 mg oral alendronate weekly and 600 mg calcium and 125 IU of vitamin D daily. Thirteen tagSNPs in NF-*κ*B1 (rs28362491, rs3774937, rs230521, rs230510, and rs4648068), RELA (rs7119750, rs11820062), and NLRC5 (rs289747, rs1566439, rs1684575, rs289726, rs289723, and rs41383) were chosen from the NCBI Locus Link and HapMap and genotyped individually. Genetic variation in these genes and the corresponding therapeutic response to alendronate treatment were analyzed.

**Results:**

Among the 13 tagSNPs, rs289747 was significantly correlated with the BMD change rate at the femoral neck (*P*=0.048). This significance no longer existed after Bonferroni correction. We then performed principal component analysis (PCA) and found NLRC5 (rs289747 and rs1566439) were strongly correlated with alendronate efficacy in femoral phenotypes and were major components of BMD change values, particularly total hip and intertrochanteric phenotypes. Furthermore, the PLINK linear regression GLM model revealed that haplotype TT of RELA (rs7119750 and rs11820062) and ICCTA of NF-*κ*B1 (rs28362491, rs3774937, rs230521, rs230510, and rs4648068) were associated with BMD of the total hip among each haplotype after 1 yr of treatment.

**Conclusion:**

The NF*-κ*B1, RELA, and NLRC5 genetic variations affect the therapeutic response of alendronate treatment for postmenopausal osteoporosis.

## 1. Introduction

Osteoporosis is a systemic bone disease characterized by a decrease in bone content, degradation of bone microstructure, and decrease in bone strength, resulting in bone brittleness and fracture susceptibility [2–4]. Pain, fracture, and spinal deformity are the most common symptoms. With the aggravating trend of an aging population worldwide, osteoporosis is becoming a global public health issue and one of the leading causes of disability and death [5, 6] in older individuals.

Osteoporosis and bone remodeling are inextricably linked. Bone remodeling comprises the two processes of bone resorption and bone creation, both of which maintain bone mass and bone balance. The function of osteoclasts and osteoblasts becomes imbalanced when the dynamic equilibrium of bone rebuilding is disrupted. The pace of bone reduction exceeds the rate of bone production, causing an imbalance in bone metabolism and a decrease in net bone volume and bone density, leading to bone loss and osteoporosis [7, 8].

Bisphosphonates such as alendronate sodium, zoledronate sodium, risedronate sodium, and ibandronate sodium are currently the first-line treatment of osteoporosis [9, 10]. Bisphosphonates are efficient osteoclast inhibitors that help to restore the equilibrium between bone resorption and creation. Although bisphosphonate medication can increase BMD and lower the incidence of fracture [5, 11, 12], there are still individual differences in drug efficacy. Because genetic variables may explain this occurrence, identifying the indications for bisphosphonate therapy from gene targeting is of tremendous therapeutic importance.

With the advancement of molecular biology and genetic research, various genes have been discovered that have a significant impact on osteoporosis treatment. Previously, we researched the mevalonate pathway [13] and sclerostin (SOST) gene [14] to test the relationship between their polymorphisms and the variance of clinical effects of alendronate. Studies have shown the transcription factor NF-*κ*B regulates cell death and survival in response to a variety of genotoxic and inflammatory stimuli [15, 16]. It also was shown to play a key role in the regulation of bone metabolism, which can reduce bone formation and enhance bone restoration [1]. Therefore, NF-*κ*B is likely to be an important genetic factor affecting the therapeutic effect of alendronate.

We investigated the associations between genetic variations in NF-*κ*B and the therapeutic response to alendronate therapy in this study. BMD was measured at the beginning of treatment and 1 yr after. The goal of this study was to investigate the relationship between genetics and medicine, which can be used to optimize and personalize pharmacological therapy in osteoporosis patients.

## 2. Materials and Methods

### 2.1. Subjects

The Osteoporosis and Bone Disease Outpatient Clinic at Shanghai Jiao Tong University Affiliated Sixth People's Hospital recruited all participants. Some participants were drawn from our earlier pharmacogenomics studies. All participants were postmenopausal Han Chinese women from the Shanghai area who had primary osteoporosis or osteopenia and had experienced no spontaneous menstruation for at least 1 yr [13].

We used the following selection criteria [13]: natural menopause after 40 yrs of age and low BMD in the posterior-anterior L1-4, the femoral neck, or the total hip, which is defined as at least 1.0 SD below the peak mean bone density of healthy young women.

Treatment decisions in women with osteopenia were based on the significant risk of osteoporotic fractures. We evaluated participants for a high risk of fracture according to the following: history of parental hip fractures; previous low trauma fracture at humerus or radius; 10 yr probability of hip fracture ≥3%; or a major osteoporotic fracture (clinical spine, hip, forearm, and humerus fracture) ≥3% using the Fracture Risk Assessment Tool (FRAX®).

The exclusion criteria [13, 14] were as follows: (i) history of chronic renal disease manifested by an endogenous creatinine clearance of <35 ml/min; (ii) acute inflammation of the gastrointestinal tract (e.g., gastritis and ulcerations); (iii) esophagitis or certain malformations and malfunctions of the esophagus (e.g., strictures and achalasia); (iv) proton-pump inhibitor usage along with alendronate treatment; (v) inability to stand, walk, or sit for 30 min after oral administration of alendronate; (vi) hypersensitivity to alendronate or another ingredient in the therapeutic compound; (vii) hypocalcemia, with a serum calcium (Ca) <2.08 mmol/l or hypophosphatemia, with a serum phosphorus (P) <0.80 mmol/l; (viii) increased serum parathyroid hormone (PTH) levels, with reference values of 15–65 pg/ml; (ix) patients with a serum level of 25 (OH) *D* <20 ng/mL; (x) serious residual effects of cerebral vascular disease; (xi) diabetes mellitus, except for adult asymptomatic hyperglycemia controlled by diet; (xii) chronic liver disease or alcoholism; (xiii) 12 weeks of corticosteroid therapy at pharmacologic levels; (xiv) 6 months of treatment with anticonvulsant therapy; (xv) evidence of other metabolic or inherited bone diseases such as hyperparathyroidism, hypoparathyroidism, Paget's disease of the bone, osteomalacia, or osteogenesis imperfecta; (xvi) rheumatoid arthritis or collagen disease; (xvii) significant disease of any endocrine organ that would affect bone mass such as Cushing's syndrome or hyperthyroidism; (xviii) any neurological or musculoskeletal condition that would be a nongenetic cause of low bone mass; (xix) a body mass index (BMI) <18 kg/m^2^ or >30 kg/m^2^; and (xx) any previous treatment with bisphosphonate, sodium fluoride, calcitonin, a selective estrogen receptor modulator, strontium ranelate, or the recombinant form of PTH or current use of hormone replacement therapy.

A total of 455 women took part in this study. For a period of 1 yr, all participants were given 70 mg of alendronate once weekly and 600 mg of calcium and 125 IU of vitamin D daily. The ethics committee at Shanghai Jiao Tong University's Affiliated Sixth People's Hospital authorized this study.

### 2.2. BMD Measurements

All patients had their bone mineral density (BMD) measured using a dual-energy X-ray absorptiometry densitometer (GE Healthcare, Madison, WI, USA) at baseline and 1 yr following treatment. The measurements were taken at the lumbar vertebrae 1–4 (L1–L4), the left femoral neck, and the total hip. We analyzed the data with the Prodigy encore program (ver. 6.70, standard-array mode; GE Healthcare). Double X-ray absorptiometry (DEXA) measured coefficients of variation for L1–L4, the femoral neck, and the total hip were 1.39%, 2.22%, and 0.70%, respectively [17]. Based on phantom measurements repeated weekly, the long-term reproducibility of the DEXA instrument was 0.45%. We measured BMD before and after the 1 yr therapy.

### 2.3. SNP Selection and Genotyping

We chose 13 tagSNPs in NF-KB1 (rs28362491, rs3774937, rs230521, rs230510, and rs4648068), RELA (rs7119750, rs11820062), and NLRC5 (rs289747, rs1566439, rs1684575, rs289726, rs289723, and rs41383) from NCBI Locus Link (https://www.ncbi.nlm.nih.gov/gene) and HapMap (https://hapmap.ncbi.nlm.nih.gov) based on the following criteria: (i) minor allele frequency (MAF) ≥0.05 and (ii) *r*2 ≥0.8.

We obtained blood samples from the participants and isolated genomic DNA from peripheral blood leukocytes using conventional phenol-chloroform extraction procedures. For genotyping, the high-throughput SNaPshot technique (Applied Biosystems, Foster City, California, USA) was used. The *χ*^2^ test was used to test the genotype frequency against the Hardy–Weinberg equilibrium (HWE) to detect genotype errors.

### 2.4. Statistical Analysis

Our calculation showed a sample size of at least 426 was needed to achieve 80% detection on Quanto (https://hydra.usc.edu/gxe/) with a two-tailed *P*-value of 0.05. The Hardy–Weinberg equilibrium (HWE) was used to test each single nucleotide polymorphism (SNP) using the *χ*^2^ test. Haploview 4.2 was used to assess the structure of the linkage disequilibrium block. The Stephens algorithm was used to construct the haplotype from population genotype data using Phase 2.0.2 software.

The Lewontin coefficient D' and linkage disequilibrium (LD) *r*^2^ between all biallelic pairs were checked. PLINK was used for quality control filtering and haplotype-related tests.

In the subsequent analysis, the HWE test for SNPs with a detection rate of <75% and the HWE test with *P*-values <0.0001 were excluded. Continuous variables with normal distribution are expressed as mean ± standard deviation. The paired *t*-test was used to compare the BMD values of L1–4, the femoral neck, and the total hip before and after alendronate treatment.

We used SPSS 22.0 to analyze the data. The response to the treatment was estimated by the change rate of BMD. The linear ADD model in PLINK was used to test the differences in BMD between the baseline and % change after 1 yr of treatment.

According to the least significant change (LSC) in BMD for all phenotypes, participants were separated into two groups: responders and nonresponders. We used the ADD logistic regression model in PLINK to analyze the association between the three groups of genotypes (NF-*κ*B, RELA, and NLRC5) and determine their possible response after treatment.

As there may be strong correlations between several original phenotypes, thus causing biases in the results of analysis, we further use principal component analysis (PCA) and MultiPhen analysis to correct for these errors. MultiPhen analysis is a new method for carrying out genome-wide association studies on several phenotypes in a short period by discovering the linear combination of the phenotypes most related to the genotype at each SNP [18]. This analysis reverses the regression in such a way that the SNP is regressed on the phenotype, rather than the phenotype on the SNP, which is what happens normally. These approaches have the potential to improve statistical power.

To explore influencing factors in more detail, the generalized linear model (GLM) function in PLINK was used to investigate the difference in baseline BMD between haplotype groups and the rate of change 1 yr after therapy. The GLM logistic regression model in PLINK was used to investigate the relationship between the haplotype groups and their probability of becoming responders.

Because BMD changes with age, height and weight have an important effect on bone density. Thus, we adjusted all data for participant age and BMI as covariates to remove the influence of these two variables.

In this study, a value of *P*=0.048 was defined as nominally significant and the Bonferroni-corrected *P*-value statistical significance threshold was 0.008 for alleles and 0.025 for haplotypes.

## 3. Results

### 3.1. Basic Characteristics of All Participants

Our study comprised 455 women who all received 1 yr of alendronate medication and performed biochemical assessments and BMD measurements at baseline and after 1 yr of therapy.

The average baseline age, height, weight, and BMI were 66.74 ± 8.37 yrs, 1.54 ± 0.06 cm, 54.72 ± 8.38 kg, and 23.14 ± 3.10 kg/m^2^, respectively. The average BMD of L0031–4, femoral neck, trochanter, intertrochanter, and total hip were 0.81 ± 0.15, 0.67 ± 0.10, 0.55 ± 0.10, 0.84 ± 0.14, and 0.72 ± 0.11 g/cm^2^, respectively (Table 1).

### 3.2. The Information of 13 tagSNPs

We successfully genotyped 13 tagSNPs in all participants and none of the SNPs failed the minor allele frequency test (MAF <0.01). In addition, all tagSNPs in the NF-*κ*B signaling pathway were in line with the HWE principle (Table 2).

### 3.3. The Relationship between Genetic Polymorphism and Alendronate Treatment Response

At baseline, there was no significant difference in BMD between the different genotype groups of L1–4, femoral neck, trochanter, intertrochanter, and total hip after the Bonferroni correction (*P* > 0.004) (Table 3).

After 1 yr of alendronate treatment, BMD increased significantly, as follows: L1–4, 4.73 ± 5.34%; neck, 2.06 ± 4.47%; total hip, 2.00 ± 3.49%; intertrochanter, 1.98 ± 3.94%; and trochanter, 3.40 ± 5.78%. Furthermore, we used PLINK software to analyze the difference in the change rate of BMD within selected tagSNPs by linear regression.

Among the 13 tagSNPs identified in this study, rs289747 of NLRC5 was significantly correlated with the femoral neck BMD change rate (% change) before Bonferroni correction (*P*=0.048) (Table 4). However, after Bonferroni correction, this significant correlation no longer existed.

To quantitatively analyze the difference in the treatment efficacy for each of the tagSNPs, all participants were divided according to LSC into two groups: responders and nonresponders. In this study, 3% of the BMD change rate was chosen as the LSC. We used the PLINK software logistic regression model to analyze the differences in treatment efficacy within selected tagSNPs.

Among the 13 tagSNPs, rs1684575 of NLRC5 was significantly correlated with L1–4 response, and rs41383 of NLRC5 was significantly correlated with the intertrochanter response (Table 5). However, after the Bonferroni correction, there was no significant difference between the groups either.

Since both the BMD change rate and the BMD response are no longer significant after Bonferroni correction, we consider other methods to analyze the experimental data.

### 3.4. PCA Analysis and MultiPhen Analysis

Considering that the efficacy of alendronate on different phenotypes may be correlated, the significance of the above analysis results has been affected. Therefore, we performed further analysis to rule out the effect of this correlation.

**Figure 1:**
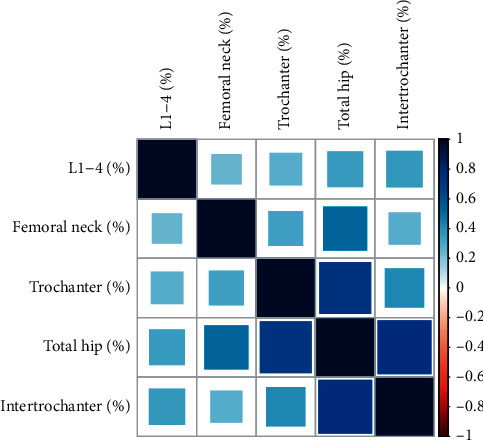
The correlation heat map of five bone density phenotypes.

As expected, we found that the femoral neck, trochanter, total hip, and intertrochanter were significantly correlated in BMD change rate (Figure 1). Hence, we subsequently investigated the relationships between SNPs and multiple phenotypes using principal component analysis (PCA) and MultiPhen analysis.

**Figure 2:**
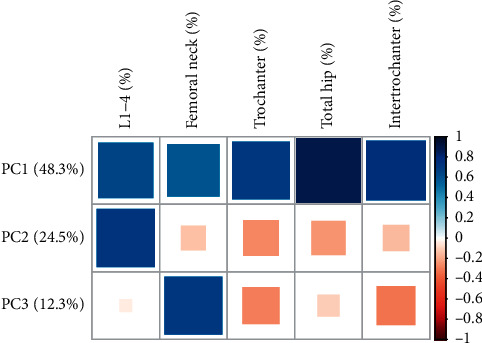
The loadings of the first three principal components for five bone density phenotypes. Blue means positive correlation, while pink means negative correlation.

Within the five phenotypes, we obtained the first principal component (PC1), second principal component (PC2), and third principal component (PC3) by PCA analysis, which accounted for 48.3%, 24.5%, and 12.3% of the variance in the original phenotypes, respectively (Figure 2).

Following these studies, we examined the relationships between SNPs and principal components. The findings revealed that, in the BMD change rate (Table 6) and in the response (Table 7), both rs289747 and rs1566439 of NLRC5 had a strong relationship with PC3, to which the femoral neck, trochanter, and intertrochanter contributed. These results are consistent with the MultiPhen analysis results (Table 6).

**Figure 3:**
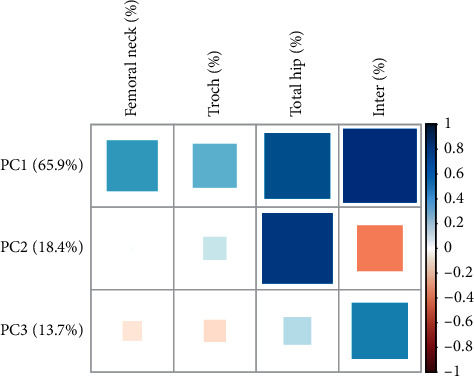
The loadings of the first three principal components for four bone density phenotypes. Blue means positive correlation, while pink means negative correlation.

Considering the differences in the composition of the lumbar spine and femur, the role of principal component analysis may be weakened. We used the four phenotypes, including femoral neck, trochanter, total hip, and intertrochanter, to extract PC1, PC2, and PC3, which accounted for 65.9%, 18.4%, and 13.7% of the variance in the original phenotypes, respectively (Figure 3). We found the rs289747 and rs1566439 of NLRC5 were significantly correlated with PC2, which was dominantly contributed by the total hip and intertrochanter, in terms of BMD response (Table 8). The results showed that rs289747 and rs1566439 were strongly linked with the pharmacological effects in femoral phenotypes and major components of BMD change values, particularly the total hip and intertrochanter phenotypes.

### 3.5. The Relationship between Haplotypes and Alendronate Treatment Response

In order to expand the research to a more granular level, we further analyzed different haplotype combinations of each SNP.

The three kinds of genes of the NF-*κ*B signaling pathway were analyzed by PLINK software and 12 haplotypes were obtained. GLM was used to analyze the differences in baseline BMD values among haplotypes adjusted for age and BMI. The results showed that the haplotype CCT of NLRC5 (rs289747, rs1566439, and rs1684575) was significantly correlated with the baseline BMD of trochanter, intertrochanter, and total hip measurements (Table 9).

We further analyzed the difference in the change rate of BMD (adjusted for age BMI and baseline BMD) among each haplotype after 1 yr of treatment. The linear regression ADD model of PLINK software was used to analyze the difference in the percentage increase of BMD in each part of each SNP with different haplotypes after 1 yr of treatment. We found that the haplotype TT of RELA (rs7119750 and rs11820062) was substantially linked with the BMD of total hip (Table 10).

Finally, in order to examine the difference in treatment efficacy of the haplotype, all participants were split into two groups of responders and nonresponders, based on LSC (3% as before), as was done with the prior criterion. Following this, we used PLINK software in a logistic regression model to examine the difference in treatment efficacy in each site after 1 yr of treatment among the 12 haplotypes of the three selected tagSNPs.

Bonferroni correction was performed on the results (Table 11). The haplotypes TT of RELA (rs7119750 and rs11820062) and ICCTA of NF-*κ*B1 (rs28362491, rs3774937, rs230521, rs230510, and rs4648068) were significantly correlated with the treatment efficacy of L1-4 and the total hip, respectively.

## 4. Discussion

Alendronate is a safe and effective bone resorption inhibitor for the treatment of osteoporosis [19–21]. However, many issues with its use remain unresolved, such as individual variances in treatment effectiveness. As a result, more clinical trials are required to support the use of alendronate to prevent bone loss and osteoporosis [22–24]. Osteoporosis is a multifaceted illness affected by genetic and environmental variables, with a high degree of genetic determinacy [25]. We performed this pharmacogenomic research to determine if genetic variables contribute to individual variances in response to alendronate administration.

The genetic study of osteoporosis etiology has become a hot topic in recent years, but the functions of its associated genes are still unknown. As a result, the study of osteoporosis candidate genes, such as genetic polymorphisms, to find the genetic mechanism of osteoporosis is important. Genetic polymorphism research might provide evidence to help guide early and effective prevention and clinical screening of high-risk groups for osteoporosis or bone fracture [2]. Nearly 100 osteoporosis-related genes have been studied so far, with the majority involving hormones that regulate calcium balance and their receptors, growth factors and their receptors, bone matrix, and sex hormones and their receptors [26].

The NF-*κ*B signaling pathway is important in the control of bone metabolism because it can limit bone production while increasing bone regeneration [1]. Furthermore, animals lacking the NF-*κ*B p65 subunit displayed abnormal osteoclast development and osteolysis [27]. NF-*κ*B activation stimulates the release of inflammatory molecules such as IL-6 and TNF-*α*. Downregulation of NF-*κ*B p65 expression might drastically reduce senescence-related secretion and alleviate osteoporosis in mice [28]. These findings suggest that the NF-*κ*B signaling pathway may be crucial in controlling the effects of DNA damage on bone metabolism. These findings suggest that an NF-*κ*B inhibitor is a novel potential medication that can both impede bone repair and induce bone growth. Study of NF-*κ*B inhibitors may further the treatment of osteoporosis in older persons, as well as osteoporosis induced by radiation or DNA damage repair problems. These findings are consistent with our previous research, which found that rs289747 was substantially associated with the femoral neck change rate before Bonferroni correction (*P*=0.048) among the 13 tagSNPs in this study.

With the advancement of molecular biology and genetics, researchers have discovered various genes connected to the NF-*κ*B signaling pathway that have a significant influence on osteoporosis therapy [15, 16]. In response to genotoxic and inflammatory stimuli, NF-*κ*B enters the nucleus and stimulates transcription of several target genes that govern cellular stress, including cell silencing and apoptosis [29]. Some strategies, such as decoy oligodeoxynucleotides [17], resveratrol [30], celastrol [27], and glycyrrhizic acid [31], inhibit NF-*κ*B, thereby preventing osteoporosis by inhibiting bone resorption. Taken together, these findings indicate that NF–B inhibitors are a novel potential pharmacotherapy with a bright future in the treatment of osteoporosis from multiple etiologies. Because the number of studies on the NF-*κ*B signaling route in osteoporosis is insufficient, we evaluated the association between NF-*κ*B signaling pathway polymorphism and the efficacy of osteoporosis treatment.

The results of many studies have shown that there are many factors that affect bone mineral density. A considerable amount of literature has studied the influencing factors including genes and the environment [32]. The research on the influence of age and BMI on bone mineral density has revealed that these two factors have a significant impact on bone density [33–35]. Therefore, in order to avoid the influence of these two factors on this study, we adjusted the data with age and BMI in the statistical analysis as in our previous study [13, 14].

In this work, we discovered connections between several phenotypes and we subsequently investigated the links between SNPs and numerous phenotypes using PCA and MultiPhen analysis. We found rs289747 and rs1566439 were strongly linked with the pharmacological effects of femoral phenotypes and major components of BMD change values, particularly in the total hip and intertrochanter phenotypes.

Furthermore, we explored the connection between the NF-*κ*B signaling pathway haplotype and alendronate therapy response. After controlling for age and BMI, the findings revealed that there was no significant variation in the BMD change rate of any area across different SNP genotypes. After 1 yr of therapy, the PLINK-generated linear regression GLM revealed that the haplotypes ICCTA of NF-ΚB1 and TT of RELA were significantly correlated with the treatment efficacy alendronate in L1–4 and the total hip, respectively. Based on the results of this study combined with our previous results [13, 14], we conclude that NF-*κ*B is one of many genes that affect the therapeutic effect of alendronate. We will research the connection between these genes and their joint influence on the therapeutic effect of alendronate in future studies.

In conclusion, this study revealed that the NF-*κ*B signaling pathway, including the NF-*κ*B1 RELA and NLRC5, may be implicated in alendronate's success in the treatment of osteoporosis, which can provide an early prediction of alendronate's efficacy in the treatment of osteoporosis.

## Figures and Tables

**Table 1:** Basic characteristics of 455 postmenopausal women.

Characteristics	Mean	SD	Min	Max
Age	66.74	8.37	48	90
Height	1.54	0.06	1.35	1.73
Weight	54.72	8.38	27	86
BMI	23.14	3.10	14.18	34.28
L1-4	0.81	0.15	0.39	1.45
Neck	0.67	0.10	0.42	1.09
Trochanter	0.55	0.10	0.27	1.12
Intertrochanter	0.84	0.14	0.33	1.25
Total	0.72	0.11	0.41	

**Table 2:** Information of the 13 SNPs in this study.

SNP	CHR	Position	Gene	SNP	Major allele	Minor allele	MAF	HWE P
rs28362491	4	102500997	NFKB1	5'-flanking	C	CATTG	0.397	0.597
rs3774937	4	102513096	NFKB1	intron1	T	C	0.350	0.679
rs230521	4	102542171	NFKB1	intron5	G	C	0.426	0.414
rs230510	4	102555009	NFKB1	intron5	A	T	0.499	0.767
rs4648068	4	102597148	NFKB1	intron14	G	A	0.373	0.556
rs7119750	11	65655120	RELA	intron10	C	T	0.370	0.754
rs11820062	11	65662465	RELA	intron1	C	T	0.419	0.394
rs289747	16	56990026	NLRC5	5'-flanking	C	T	0.270	0.859
rs1566439	16	56990750	NLRC5	5'-flanking	C	T	0.413	0.474
rs1684575	16	57023707	NLRC5	intron3	G	T	0.301	0.919
rs289726	16	57040539	NLRC5	intron15	T	C	0.377	0.988
rs289723	16	57046616	NLRC5	nonsynon_exon21	A	C	0.165	0.719
rs41383	16	57077090	NLRC5	Intron39	C	T	0.347	0.992

Note: MAF： minimum allele frequency； HWE： Hardy–Weinberg test.

**Table 3:** The association between 13 SNPs and BMD of baseline.

Gene	SNP	L1-4 (%)		L2-4 (%)		Femoral neck (%)		Troch (%)		Inter (%)		Total hip (%)	
		Beta	*P*	Beta	*P*	Beta	*P*	Beta	*P*	Beta	*P*	Beta	*P*
NFKB1	rs28362491	−9.7*E* – 03	0.376	−1.5*E* – 03	0.914	−8.3*E* – 03	0.226	−1.1*E* – 02	0.126	−4.8*E* – 03	0.492	2.1*E* – 03	0.827
	rs3774937	−9.5*E* – 03	0.391	−7.4*E* – 03	0.604	−1.3*E* – 02	0.062	−1.7*E* – 02	0.025	−9.3*E* – 03	0.189	−2.9*E* – 03	0.767
	rs230521	−7.9*E* – 03	0.468	−6.6*E* – 03	0.630	−9.8*E* – 03	0.153	−1.2*E* – 02	0.104	−5.4*E* – 03	0.438	3.4*E* – 03	0.720
	rs230510	2.*E* – 03	0.799	−3.1*E* – 03	0.819	6.3*E* – 03	0.344	5.2*E* – 03	0.463	6.6*E* – 04	0.922	−7.6*E* – 03	0.407
	rs4648068	−1.1*E* – 02	0.307	−1.1*E* – 02	0.415	−1.2*E* – 02	0.078	−1.4*E* – 02	0.064	−8.1*E* – 03	0.249	−1.1*E* – 03	0.905

RELA	rs7119750	2.0*E* – 03	0.859	−3.1*E* – 03	0.825	1.3*E* – 04	0.985	−7.7*E* – 03	0.300	−2.6*E* – 03	0.706	−1.8*E* – 03	0.854
	rs11820062	−5.8*E* – 03	0.589	−8.9*E* – 03	0.521	−3.9*E* – 03	0.571	5.0*E* – 04	0.945	−1.3*E* – 03	0.850	−2.2*E* – 03	0.811

NLRC5	rs289747	−1.0*E* – 03	0.932	−9.7*E* – 03	0.516	−6.9*E* – 03	0.341	−1.6*E* – 02	0.042	−1.5*E* – 02	0.047	−2.9*E* – 02	0.004
	rs1566439	−5.7*E* – 04	0.958	−9.6*E* – 03	0.479	−5.1*E* – 03	0.452	−1.4*E* – 02	0.052	−1.0*E* – 02	0.143	−2.3*E* – 02	0.011
	rs1684575	−1.1*E* – 02	0.315	−9.7*E* – 03	0.477	1.2*E* – 03	0.864	9.4*E* – 03	0.201	3.1*E* – 03	0.655	−3.1*E* – 03	0.739
	rs289726	−1.8*E* – 03	0.863	−3.8*E* – 04	0.977	5.5*E* – 03	0.406	1.3*E* – 02	0.075	6.6*E* – 03	0.331	2.4*E* – 03	0.796
	rs289723	−6.2*E* – 05	0.996	−1.2*E* – 03	0.941	−8.4*E* – 03	0.316	7.1*E* – 03	0.421	−4.2*E* – 04	0.961	−3.7*E* – 03	0.743
	rs41383	−1.6*E* – 03	0.883	3.2*E* – 04	0.981	1.2*E* – 02	0.090	9.5*E* – 03	0.190	6.1*E* – 03	0.387	6.5*E* – 03	0.486

**Table 4:** The association between 13 SNPs and the % change in BMD.

Gene	SNP	L1-4 (%)		Femoral neck (%)		Trochanter (%)		Intertrochanter (%)		Total hip (%)	
		Beta	*P*	Beta	*P*	Beta	*P*	Beta	*P*	Beta	*P*
NFKB1	rs28362491	−0.040	0.919	−0.054	0.867	−0.210	0.636	0.029	0.924	0.193	0.443
	rs3774937	−0.326	0.419	−0.090	0.784	−0.297	0.509	−0.004	0.990	0.148	0.560
	rs230521	−0.082	0.837	0.066	0.839	0.048	0.914	−0.030	0.920	0.231	0.356
	rs230510	0.173	0.654	−0.105	0.739	0.089	0.834	−0.080	0.782	−0.178	0.465
	rs4648068	−0.350	0.382	0.027	0.936	0.072	0.871	−0.026	0.931	0.198	0.433

RELA	rs7119750	−0.158	0.693	−0.200	0.540	0.368	0.411	−0.277	0.363	−0.036	0.886
	rs11820062	−0.148	0.706	0.113	0.727	−0.295	0.501	−0.369	0.216	−0.220	0.379

NLRC5	rs289747	−0.124	0.775	−0.695	**0.048**	0.534	0.261	0.511	0.114	0.178	0.509
	rs1566439	−0.135	0.732	−0.589	0.069	0.620	0.154	0.407	0.169	0.190	0.444
	rs1684575	0.251	0.525	−0.138	0.674	−0.004	0.993	0.121	0.688	0.040	0.874
	rs289726	−0.082	0.828	0.170	0.590	0.490	0.254	−0.057	0.846	0.122	0.618
	rs289723	−0.159	0.741	0.414	0.297	0.012	0.982	0.191	0.597	0.180	0.557
	rs41383	−0.046	0.908	0.195	0.551	0.754	0.084	0.306	0.304	0.239	0.342

Significant association between 13 SNPs and the % change in BMD before Bonferroni correction (*P* < 0.05) values are shown in bold.

**Table 5:** The association between 13 SNPs and the BMD response.

Gene	SNP	L1-4			Femoral neck			Trochanter (%)			Total hip (%)			Intertrochanter (%)			
		OR	95% CI	*P*	OR	95% CI	*P*	OR	95% CI	*P*	OR	95% CI	*P*	OR	95% CI		*P*
NFKB1	rs28362491	0.936	0.804–1.091	0.666	0.997	0.858–1.16	0.987	0.894	0.767–1.04	0.459	1.03	0.887–1.195	0.845	0.975	0.835–1.139		0.872
	rs3774937	0.778	0.666–0.909	0.106	1.066	0.916–1.241	0.675	0.889	0.762–1.038	0.447	1.087	0.935–1.264	0.578	0.936	0.799–1.097		0.676
	rs230521	0.866	0.743–1.009	0.347	1.063	0.914–1.235	0.687	0.947	0.814–1.102	0.718	1.117	0.963–1.295	0.458	0.999	0.856–1.167		0.996
	rs230510	1.116	0.962–1.294	0.461	0.88	0.76–1.018	0.38	1.012	0.875–1.171	0.934	0.875	0.757–1.011	0.355	0.989	0.851–1.148		0.939
	rs4648068	0.75	0.643–0.876	0.063	1.073	0.922–1.249	0.641	0.983	0.845–1.145	0.912	1.148	0.988–1.333	0.358	0.955	0.817–1.116		0.769

RELA	rs7119750	0.979	0.839–1.141	0.888	0.904	0.777–1.052	0.505	1.105	0.948–1.288	0.516	1.049	0.904–1.218	0.749	0.889	0.76–1.041	0.455
	rs11820062	0.983	0.845–1.144	0.91	1.171	1.008–1.359	0.291	1	0.86–1.162	0.998	0.775	0.667–0.9	0.088	0.792	0.678–0.925		0.134

NLRC5	rs289747	1.102	0.932–1.303	0.561	0.728	0.615–0.862	0.06	1.094	0.929–1.288	0.581	1.135	0.968–1.332	0.427	1.341	1.135–1.584		0.078
	rs1566439	1.085	0.932–1.263	0.592	0.903	0.777–1.049	0.496	1.21	1.042–1.407	0.204	1.086	0.937–1.259	0.578	1.213	1.04–1.414		0.209
	rs1684575	1.486	1.269–1.741	0.012	1.061	0.913–1.233	0.693	1.034	0.889–1.203	0.825	0.864	0.744–1.005	0.333	0.955	0.817–1.115		0.765
	rs289726	1.232	1.063–1.429	0.158	1.289	1.114–1.491	0.081	1.179	1.017–1.367	0.265	1.003	0.869–1.159	0.982	1.003	0.863–1.166		0.982
	rs289723	1.3	1.073–1.574	0.171	1.372	1.146–1.643	0.079	1.21	1.008–1.453	0.296	1.166	0.974–1.396	0.393	1.058	0.879–1.273		0.762
	rs41383	1.037	0.891–1.208	0.811	1.142	0.983–1.327	0.375	1.019	0.877–1.183	0.901	1.04	0.895–1.208	0.793	1.404	1.203–1.638		0.028

Significant association between 13 SNPs and the BMD response (*P* < 0.05) values are shown in bold.

**Table 6:** The association between 13 SNPs and the PCs and the % change in BMD change.

Gene	SNP	PC1		PC2		PC3		MultiPhen	
		Beta	*P*-value	Beta	*P*-value	Beta	*P*-value	*P*-value of OP	*P*-value of PCs
NFKB1	rs28362491	0.0109	0.9303	−0.0087	0.9212	−0.0041	0.9480	0.2349	0.7776
	rs3774937	−0.0415	0.7406	−0.0671	0.4522	−0.0023	0.9710	0.7426	0.8066
	rs230521	0.0345	0.7797	−0.0308	0.7259	0.0090	0.8851	0.2671	0.7073
	rs230510	−0.0157	0.8955	0.0629	0.4590	−0.0173	0.7751	0.8264	0.8832
	rs4648068	−0.0039	0.9750	−0.0968	0.2746	0.0006	0.9928	0.7280	0.7709

RELA	rs7119750	−0.0590	0.6346	−0.0650	0.4623	−0.0430	0.4938	0.2007	0.3372
	rs11820062	−0.0816	0.5076	0.0565	0.5191	0.0722	0.2460	0.8814	0.8597

NLRC5	rs289747	0.0570	0.6688	−0.0523	0.5805	−0.2063	0.0021	0.0338	0.0111
	rs1566439	0.0520	0.6710	−0.0824	0.3442	−0.1853	0.0027	0.2176	0.0245
	rs1684575	0.0367	0.7674	0.0577	0.5140	−0.0373	0.5526	0.5578	0.5257
	rs289726	0.0513	0.6688	−0.0473	0.5797	0.0101	0.8686	0.6430	0.9621
	rs289723	0.0535	0.7229	−0.0978	0.3630	0.0661	0.3872	0.8199	0.8866
	rs41383	0.1586	0.2012	−0.0371	0.6753	−0.0300	0.6330	0.2967	0.8344

Significant association between 13 SNPs and the PCs and the % change in BMD change (*P* < 0.05) values are shown in bold.

**Table 7:** The association between 13 SNPs and the PCs within five phenotypes of BMD response.

Gene	SNP	PC1			PC2			PC3		
		OR	95% CI	*P*	OR	95% CI	*P*	OR	95% CI	*P*
NFKB1	rs28362491	0.889	0.769–1.028	0.416	0.970	0.839–1.121	0.832	0.949	0.822–1.096	0.715
	rs3774937	0.944	0.815–1.093	0.694	0.843	0.727–0.977	0.246	0.967	0.836–1.118	0.816
	rs230521	0.939	0.813–1.085	0.662	0.903	0.782–1.044	0.483	1.023	0.886–1.181	0.875
	rs230510	1.132	0.984–1.302	0.378	1.116	0.97–1.284	0.433	0.999	0.869–1.148	0.993
	rs4648068	0.959	0.829–1.11	0.775	0.838	0.724–0.97	0.228	1.019	0.882–1.178	0.894

RELA	rs7119750	1.129	0.976–1.306	0.403	0.881	0.762–1.019	0.385	0.885	0.766–1.023	0.399
	rs11820062	0.864	0.748–0.998	0.312	1.179	1.02–1.362	0.254	1.009	0.875–1.165	0.948

NLRC5	rs289747	0.884	0.756–1.033	0.429	1.020	0.873–1.192	0.899	0.644	0.55–0.755	0.005
	rs1566439	0.884	0.765–1.02	0.389	0.927	0.803–1.07	0.597	0.747	0.647–0.862	0.042
	rs1684575	0.871	0.753–1.008	0.344	1.011	0.874–1.169	0.939	1.019	0.882–1.177	0.897
	rs289726	1.023	0.889–1.177	0.872	1.008	0.876–1.16	0.956	1.112	0.967–1.279	0.448
	rs289723	0.947	0.794–1.13	0.759	0.944	0.791–1.128	0.747	1.265	1.06–1.51	0.183
	rs41383	1.203	1.039–1.391	0.206	1.032	0.892–1.193	0.830	1.002	0.868–1.158	0.987

Significant association between 13 SNPs and the PCs within five phenotypes of BMD response (*P* < 0.05) values are shown in bold.

**Table 8:** The association between 13 SNPs and the PCs within four phenotypes and the BMD response.

Gene	SNP	PC1			PC2			PC3		
		OR	95% CI	*P*-value	OR	95% CI	*P*-value	OR	95% CI	*P*-value
NFKB1	rs28362491	1.021626	0.883–1.182	0.883236	0.956371	0.828–1.105	0.756795	0.967341	0.838–1.117	0.817612
	rs3774937	1.060885	0.915–1.229	0.688588	0.989373	0.855–1.145	0.94156	0.879019	0.76–1.017	0.377434
	rs230521	1.077348	0.932–1.246	0.607872	1.033903	0.896–1.193	0.816238	0.996556	0.863–1.15	0.980814
	rs230510	0.896616	0.779–1.032	0.438492	0.980647	0.853–1.127	0.888268	1.15232	1.002–1.325	0.309287
	rs4648068	1.099887	0.95–1.274	0.516014	1.045775	0.905–1.209	0.757285	0.937384	0.811–1.084	0.655449

RELA	rs7119750	0.942271	0.814–1.091	0.684227	0.853131	0.738–0.986	0.272774	1.100183	0.952–1.271	0.508311
	rs11820062	0.948197	0.82–1.096	0.714066	1.06157	0.92–1.225	0.676695	1.193068	1.033–1.377	0.219405

NLRC5	rs289747	1.000547	0.855–1.17	0.997216	0.637724	0.544–0.748	0.00465	1.09926	0.942–1.283	0.540538
	rs1566439	0.944905	0.818–1.092	0.694567	0.737524	0.638–0.852	0.03476	1.101492	0.955–1.27	0.497623
	rs1684575	0.8208	0.709–0.951	0.178707	1.015737	0.879–1.174	0.91391	1.159657	1.003–1.34	0.305928
	rs289726	0.975745	0.847–1.124	0.861781	1.076318	0.936–1.238	0.598353	1.250357	1.087–1.439	0.111212
	rs289723	1.085625	0.909–1.297	0.643858	1.262391	1.058–1.506	0.187237	1.05209	0.883–1.254	0.772481
	rs41383	1.064361	0.92–1.232	0.669756	0.988057	0.855–1.141	0.933651	1.065325	0.922–1.231	0.660928

Significant association between 13 SNPs and the PCs within four phenotypes and the BMD response (*P* < 0.05) values are shown in bold.

**Table 9:** The relationship between 12 haplotypes of 3 candidate genes and baseline BMD.

Gene	SNP	Haplotype	L1-4 (%)		Femoral neck (%)		Trochanter (%)		Intertrochanter (%)		Total hip (%)	
			Beta	*P*-value	Beta	*P*-value	Beta	*P*-value	Beta	*P*-value	Beta	*P*-value
NFKB1	rs28362491	CCCTG	−2.60*E* – 03	0.894	6.80*E* – 03	0.61	4.80*E* – 03	0.729	1.20*E* – 03	0.93	−5.40*E* – 03	0.766
	rs3774937	ITGAA	−7.60*E* – 03	0.603	−1.10*E* – 02	0.266	−2.20*E* – 02	0.035	−5.10*E* – 03	0.62	1.10*E* – 02	0.439
	rs230521	ICCTA	1.10*E* – 02	0.477	−9.80*E* – 03	0.347	3.30*E* – 03	0.765	−1.20*E* – 02	0.258	−2.90*E* – 02	0.039
	rs230510											
	rs4648068											

RELA	rs7119750	TT	5.90*E* – 03	0.652	−1.10*E* – 03	0.906	6.60*E* – 03	0.482	−5.30*E* – 04	0.954	2.90*E* – 03	0.813
	rs11820062	TC	2.60E–03	0.868	2.60*E* – 03	0.808	3.00*E* – 03	0.789	4.20*E* – 03	0.7	3.80*E* – 04	0.979
		CT	−9.70*E* – 03	0.507	−9.90*E* – 04	0.921	−1.10*E* – 02	0.298	−3.10*E* – 03	0.762	−3.90*E* – 03	0.772

NLRC5	rs289747	TTT	8.60*E* – 03	0.413	1.10*E* – 02	0.138	9.60*E* – 03	0.2	1.20*E* – 02	0.098	2.10*E* – 02	0.03
	rs1566439	CCT	−1.50*E* – 03	0.915	−1.60*E* – 02	0.099	−2.40*E* – 02	0.022	−2.30*E* – 02	0.02	−3.70*E* – 02	0.005
	rs1684575	TTG	−1.40*E* – 02	0.319	8.50*E* – 04	0.931	5.70*E* – 03	0.579	5.00*E* – 04	0.96	−3.60*E* – 03	0.79
	rs289726	CAT	5.00*E* – 03	0.736	−4.60*E* – 03	0.647	−6.70*E* – -03	0.526	3.40*E* – 03	0.742	3.00*E* – 03	0.824
	rs289723	CCT	−1.60*E* – 02	0.267	3.20*E* – 03	0.752	−2.40*E* – 03	0.818	−2.80*E* – 03	0.787	7.20*E* – 03	0.599
	rs41383	TCT	1.50*E* – 02	0.453	−2.20*E* – 02	0.103	−1.00*E* – 02	0.472	−1.10*E* – 02	0.417	−1.30*E* – 02	0.455

Significant association between 12 haplotypes of 3 candidate genes and baseline BMD (*P* < 0.025) values are shown in bold.

**Table 10:** The relationship between 12 haplotypes of 3 candidate genes and the % change of BMD.

Gene	SNP	Haplotype	L1-4 (%)		Femoral neck (%)		Trochanter (%)		Intertrochanter (%)		Total hip (%)	
			Beta	*P*-value	Beta	*P*-value	Beta	*P*-value	Beta	*P*-value	Beta	*P*-value
NFKB1	rs28362491	CCCTG	1.13	0.11	0.01	0.984	0.46	0.55	−0.07	0.873	0.23	0.659
	rs3774937	ITGAA	−0.13	0.808	−0.26	0.548	−0.31	0.599	0.26	0.438	0.15	0.714
	rs230521	ICCTA	−1.26	0.021	−0.06	0.891	−0.42	0.485	−0.31	0.372	−0.31	0.448
	rs230510											
	rs4648068											

RELA	rs7119750	TT	−0.01	0.983	0.45	0.241	−0.45	0.39	0.33	0.271	0.86	0.015
	rs11820062	TC	−0.12	0.828	−0.21	0.643	−0.07	0.904	−0.29	0.404	−0.68	0.106
		CT	0.12	0.819	−0.38	0.381	0.63	0.281	−0.15	0.657	−0.47	0.231

NLRC5	rs289747	TTT	0.12	0.742	0.37	0.225	−0.4	0.329	−0.13	0.594	−0.32	0.261
	rs1566439	CCT	−0.23	0.662	−0.58	0.17	0.88	0.124	0.23	0.49	0.4	0.306
	rs1684575	TTG	0.28	0.589	−0.45	0.289	−0.18	0.747	−0.05	0.886	0.2	0.614
	rs289726	CAT	0.12	0.817	−0.29	0.507	−1.02	0.08	−0.11	0.747	0.07	0.858
	rs289723	CCT	−0.21	0.691	0.05	0.916	−0.16	0.784	−0.13	0.7	−0.25	0.533
	rs41383	TCT	0.58	0.422	0.16	0.787	−1.1	0.153	−0.27	0.544	−0.66	0.207

Significant association between between 12 haplotypes of 3 candidate genes and the % change of BMD (*P* < 0.025) values are shown in bold.

**Table 11:** The relationship between 12 haplotypes of 3 candidate genes and the efficacy of alendronate after one year of treatment.

Gene	SNP	Haplotype	L1-4 (%)			Femoral neck (%)			Trochanter (%)			Intertrochanter (%)			Total hip (%)		
			OR	95%CI	*P*-value	OR	95%CI	*P*-value	OR	95%CI	*P*-value	OR	95%CI	*P*-value	OR	95%CI	*P*-value
NFKB1	rs28362491	CCCTG	1.744	1.335–2.276	0.037	1.036	0.793–1.354	0.894	1.145	0.878–1.494	0.611	0.836	0.645–1.083	0.49	1.116	0.848–1.469	0.689
	rs3774937	ITGAA	0.968	0.789–1.187	0.872	0.951	0.78–1.16	0.8	0.81	0.662–0.991	0.296	1.117	0.918–1.36	0.573	0.943	0.768–1.159	0.776
	rs230521	ICCTA	0.498	0.403–0.614	0.0009	0.895	0.727–1.102	0.594	0.943	0.765–1.163	0.78	0.88	0.717–1.08	0.533	0.898	0.724–1.114	0.617
	rs230510																
	rs4648068																

RELA	rs7119750	TT	1.049	0.875–1.257	0.792	0.873	0.731–1.043	0.445	0.847	0.708–1.014	0.356	1.21	1.016–1.443	0.277	1.574	1.305–1.898	0.015
	rs11820062	TC	0.844	0.679–1.048	0.434	1.268	1.026–1.568	0.262	1.163	0.94–1.439	0.477	0.787	0.641–0.966	0.242	0.727	0.586–0.902	0.14
		CT	1.094	0.893–1.341	0.656	0.959	0.786–1.17	0.833	1.076	0.88–1.315	0.717	0.978	0.804–1.191	0.912	0.76	0.619–0.934	0.182

NLRC5	rs289747	TTT	0.78	0.674–0.904	0.091	1.053	0.913–1.214	0.716	0.883	0.765–1.019	0.384	1.046	0.908–1.204	0.751	0.898	0.775–1.041	0.466
	rs1566439	CCT	1.077	0.881–1.317	0.711	0.67	0.55–0.816	0.042	1.18	0.968–1.438	0.403	1.155	0.952–1.4	0.457	1.36	1.11–1.665	0.13
	rs1684575	TTG	1.668	1.363–2.042	0.011	0.989	0.814–1.202	0.955	0.971	0.797–1.183	0.883	0.883	0.728–1.07	0.517	1.052	0.86–1.289	0.801
	rs289726	CAT	0.82	0.668–1.007	0.335	0.702	0.572–0.862	0.085	0.726	0.592–0.889	0.115	1.022	0.838–1.247	0.911	0.906	0.736–1.116	0.635
	rs289723	CCT	0.786	0.638–0.969	0.249	1.292	1.043–1.601	0.232	1.457	1.191–1.783	0.062	1.024	0.84–1.249	0.905	1.001	0.814–1.229	0.998
	rs41383	TCT	1.291	0.969–1.719	0.373	1.013	0.758–1.355	0.964	0.841	0.644–1.1	0.519	1.074	0.826–1.396	0.787	0.673	0.506–0.896	0.166

Significant association between 12 haplotypes of 3 candidate genes and the efficacy of alendronate after one year of treatment (*P* < 0.025) values are shown in bold.

## Data Availability

The data used to support the findings of this study are included within the article.

## References

[1] Jimi E., Fukushima H. (2016). NF-*κ*B signaling pathways and the future perspectives of bone disease therapy using selective inhibitors of NF-*κ*b. *Clinical Calcium*.

[2] Lane N. E. (2006). Epidemiology, etiology, and diagnosis of osteoporosis. *American Journal of Obstetrics and Gynecology*.

[3] Marchand D., Loshak H. (2020). *Duration of Bisphosphonate Treatment for Patients with Osteoporosis: A Review of Clinical Effectiveness and Guidelines*.

[4] Rachner T. D., Khosla S., Hofbauer L. C. (2011). Osteoporosis: now and the future. *Lancet*.

[5] Bandeira F., Dantas W., Bilezikian J. P. (2020). Controversies in the treatment of postmenopausal osteoporosis: how long to treat with bisphosphonates?. *Archives of Endocrinology and Metabolism*.

[6] Ivanova S., Vasileva L., Ivanova S., Peikova L., Obreshkova D. (2015). Osteoporosis: therapeutic options. *Folia Medica*.

[7] Komm B. S., Morgenstern D., A Yamamoto L., Jenkins S. N. (2015). The safety and tolerability profile of therapies for the prevention and treatment of osteoporosis in postmenopausal women. *Expert Review of Clinical Pharmacology*.

[8] Lane J. M., Russell L., Khan S. N. (2000). Osteoporosis. *Clinical Orthopaedics and Related Research*.

[9] Chandran T., Venkatachalam I. (2019). Efficacy and safety of denosumab compared to bisphosphonates in improving bone strength in postmenopausal osteoporosis: a systematic review. *Singapore Medical Journal*.

[10] Fan G., Zhao Q., Lu P. (2020). Comparison between teriparatide and bisphosphonates for improving bone mineral density in postmenopausal osteoporosis patients. *Medicine*.

[11] Yuan F., Peng W., Yang C., Zheng J. (2019). Teriparatide versus bisphosphonates for treatment of postmenopausal osteoporosis: a meta-analysis. *International Journal of Surgery*.

[12] Solomon C. G. (2002). Bisphosphonates and osteoporosis. *New England Journal of Medicine*.

[13] Wang C., Zheng H., He J.-W. (2015). Genetic polymorphisms in the mevalonate pathway affect the therapeutic response to alendronate treatment in postmenopausal Chinese women with low bone mineral density. *Pharmacogenomics Journal*.

[14] Wang W.-J., Fu W.-Z., He J.-W., Wang C., Zhang Z.-L. (2019). Association between SOST gene polymorphisms and response to alendronate treatment in postmenopausal Chinese women with low bone mineral density. *Pharmacogenomics Journal*.

[15] Hayden M. S., Ghosh S. (2011). NF-*κ*B in immunobiology. *Cell Research*.

[16] Kunnumakkara A. B., Shabnam B., Girisa S. (2020). Inflammation, NF-*κ*B, and chronic diseases: how are they linked?. *Critical Reviews in Immunology*.

[17] Shimizu H. (2006). B Decoy oligodeoxynucleotides ameliorates osteoporosis through inhibition of activation and differentiation of osteoclasts. *Gene Therapy*.

[18] O’Reilly P., Hoggart C. J., Pomyen Y. (2012). MultiPhen: joint model of multiple phenotypes can increase discovery in GWAS. *PLoS One*.

[19] Dawson-Hughes B. (2008). A revised clinician’s guide to the prevention and treatment of osteoporosis. *The Journal of Cinical Endocrinology and Metabolism*.

[20] Kanis J. A., Cooper C., Rizzoli R., Reginster J. Y. (2019). European guidance for the diagnosis and management of osteauoporosis in postmenopausal women. *Osteoporosis International*.

[21] Watts N. B., Adler R. A., Bilezikian J. P. (2012). Osteoporosis in men: an Endocrine Society clinical practice guideline. *Journal of Clinical Endocrinology & Metabolism*.

[22] Horikawa A., Miyakoshi N., Hongo M., Kasukawa Y., Kodama H., Shimada Y. A. (2019). A prospective comparative study of intravenous alendronate and ibandronate for the treatment of osteoporosis. *Medicine*.

[23] Fink H. A., MacDonald R., Forte M. L. (2019). Long-term drug therapy and drug discontinuations and holidays for osteoporosis fracture prevention A systematic review. *Annals of Internal Medicine*.

[24] Liu G.-F., Wang Z. Q., Liu L., Zhang B. T., Miao Y. Y., Yu S. N. (2018). A network meta‐analysis on the short‐term efficacy and adverse events of different anti‐osteoporosis drugs for the treatment of postmenopausal osteoporosis. *Journal of Cellular Biochemistry*.

[25] Kim K.-T., Lee Y.-S., Han I. (2020). The role of epigenomics in osteoporosis and osteoporotic vertebral fracture. *International Journal of Molecular Sciences*.

[26] An Y., Zhang H., Wang C. (2019). Activation of ROS/MAPKs/NF*-κ*B/NLRP3 and inhibition of efferocytosis in osteoclast-mediated diabetic osteoporosis. *The FASEB Journal : Official Publication of the Federation of American Societies for Experimental Biology*.

[27] Liu X., Cai F., Zhang Y., Yang A., Liu L. (2016). Celastrol, an NF-*κ*B inhibitor, ameliorates hypercalciuria and articular cartilage lesions in a mouse model of secondary osteoporosis. *Journal of Pharmacological Sciences*.

[28] Hu B., Wu F., Shi Z. (2019). Dehydrocostus lactone attenuates osteoclastogenesis and osteoclast‐induced bone loss by modulating NF‐*κ*B signalling pathway. *Journal of Cellular and Molecular Medicine*.

[29] Ghosh S., Hayden M. S. (2008). New regulators of NF-*κ*B in inflammation. *Nature Reviews Immunology*.

[30] Feng J., Liu S., Ma S. (2014). Protective effects of resveratrol on postmenopausal osteoporosis: regulation of SIRT1-NF-*κ*B signaling pathway. *Acta Biochimica et Biophysica Sinica*.

[31] Yin Z., Zhu W., Wu Q. (2019). Glycyrrhizic acid suppresses osteoclast differentiation and postmenopausal osteoporosis by modulating the NF-*κ*B, ERK, and JNK signaling pathways. *European Journal of Pharmacology*.

[32] Lei S. F., Deng F. Y., Li M. X., Dvornyk V., Deng H. W. (2004). Bone mineral density in elderly Chinese: effects of age, sex, weight, height, and body mass index. *Journal of Bone and Mineral Metabolism*.

[33] Doğan A., Nakipoğlu-Yüzer G. F., Yıldızgören M. T., Ozgirgin N. (2010). Is age or the body mass index (BMI) more determinant of the bone mineral density (BMD) in geriatric women and men?. *Archives of Gerontology and Geriatrics*.

[34] Zhong N., Wu X. P., Xu Z. R. (2005). Relationship of serum leptin with age, body weight, body mass index, and bone mineral density in healthy mainland Chinese women. *Clinica Chimica Acta; International Journal of Clinical Chemistry*.

[35] Looker A. C., Beck T. J., Orwoll E. S. (2001). Does body size account for gender differences in femur bone density and geometry?. *Journal of Bone and Mineral Research*.

